# 

**Published:** 1937

**Authors:** 


### CONTENTS.

| | Page |
|---|---|
| **The Evolution of a Casualty Clearing Station on the Western Front.** By Richard C. Clarke, O.B.E., M.B., F.R.C.P. | 1 |
| **The Enlarged Prostate from the point of view of Practitioner and Surgeon.** By C. Ferrier Walters, F.R.C.S. | 21 |
| **The Therapeutic Value of Altitude.** By Bernard Hudson, M.D., M.R.C.P. | 35 |
| **The Rôle of the Central Nervous System in Disease: Recent Experimental Work in the U.S.S.R.** By F. Bodman, M.D. | 41 |
| **Reviews of Books** | 64 |
| **Editorial Notes** | 78 |
| **Obituaries:** George Parker, M.A., M.D. (Cantab.), LL.D. (Bristol Hon. Causa), M.R.C.S. | 82 |
| Arthur Walbank, M.B., Ch.B. Leeds, F.R.C.S. Edin., D.O.M.S. | 90 |
| **Meetings of Societies** | 91 |
| **The Medical Library of the University of Bristol** | 92 |
| **Publications Received** | 94 |
| **Local Medical Notes** | 96 |
| **The Bristol Medical Dramatic Club** | 98 |

